# Relationships between deprivation and duration of children's emergency admissions for breathing difficulty, feverish illness and diarrhoea in North West England: an analysis of hospital episode statistics

**DOI:** 10.1186/1471-2431-12-22

**Published:** 2012-03-08

**Authors:** Richard G Kyle, Malcolm Campbell, Peter Powell, Peter Callery

**Affiliations:** 1 School of Nursing, Midwifery and Health, University of Stirling, Stirling, FK9 4LA, UK; 2 School of Nursing, Midwifery and Social Work, The University of Manchester, Manchester Academic Health Science Centre, Jean McFarlane Building, Oxford Road, Manchester, M13 9PL, UK; 3 West Suffolk Hospital, Bury St Edmunds, IP33 2QZ, UK

## Abstract

**Background:**

In the United Kingdom there has been a long term pattern of increases in children's emergency admissions and a substantial increase in short stay unplanned admissions. The emergency admission rate (EAR) per thousand population for breathing difficulty, feverish illness and diarrhoea varies substantially between children living in different Primary Care Trusts (PCTs). However, there has been no examination of whether disadvantage is associated with short stay unplanned admissions at PCT-level. The aim of this study was to determine whether differences between emergency hospital admission rates for breathing difficulty, feverish illness and diarrhoea are associated with population-level measures of multiple deprivation and child well-being, and whether there is variation by length of stay and age.

**Methods:**

Analysis of hospital episode statistics and secondary analysis of Index of Multiple Deprivation (IMD) 2007 and Local Index of Child Well-being (CWI) 2009 in ten adjacent PCTs in North West England. The outcome measure for each PCT was the emergency admission rate to hospital for breathing difficulty, feverish illness and diarrhoea.

**Results:**

23,496 children aged 0-14 were discharged following emergency admission for breathing difficulty, feverish illness and/or diarrhoea during 2006/07. The emergency admission rate ranged from 27.9 to 62.7 per thousand. There were no statistically significant relationships between shorter (0 to 3 day) hospitalisations and the IMD or domains of the CWI. The rate for hospitalisations of 4 or more days was associated with the IMD (Kendall's tau_b _= 0.64) and domains of the CWI: Environment (tau_b _= 0.60); Crime (tau_b _= 0.56); Material (tau_b _= 0.51); Education (tau_b _= 0.51); and Children in Need (tau_b _= 0.51). This pattern was also evident in children aged under 1 year, who had the highest emergency admission rates. There were wide variations between the proportions of children discharged on the day of admission at different hospitals.

**Conclusions:**

Differences between rates of the more common shorter (0 to 3 day) hospitalisations were not explained by deprivation or well-being measured at PCT-level. Indices of multiple deprivation and child well-being were only associated with rates of children's emergency admission for breathing difficulty, feverish illness and diarrhoea for hospitalisations of 4 or more days.

## Background

In the United Kingdom there has been a long term pattern of increasing rates of children's emergency admissions [1,2] and a substantial increase in short stay unplanned admissions [3] despite overall improvement in children's well-being [4]. Increasing numbers of children are admitted via Emergency Departments (ED) [1] and successive audits have identified that breathing difficulty, feverish illness and diarrhoea are the three most common medical presentations at EDs for children under 15 years old [5-7], accounting for 20%, 14% and 14% of medical attendances at a UK university hospital, respectively [7]. The emergency admission rate (EAR) per thousand population for breathing difficulty, feverish illness and diarrhoea varies substantially between children living in different Primary Care Trusts (PCTs). In Greater London the highest rate is four times greater than the lowest [8]. Deprivation may contribute to increased rates [8] and duration [9] of children's hospitalisations for breathing difficulty, feverish illness and diarrhoea because poorer environmental and housing quality may contribute to increased susceptibility, spread and exacerbation of common infectious diseases [10,11]. Material deprivation, children in need, overcrowding, houses in poor condition, homelessness and environmental factors, including air quality, were all associated with differences in EARs for acute respiratory conditions in children aged 1 or more living in Greater London [8]. However, there has been no examination of whether deprivation or child well-being is associated with short stay unplanned admissions for breathing difficulty, feverish illness and diarrhoea.

The aim of this study was to determine whether rates of emergency admission to hospital for breathing difficulty, feverish illness and diarrhoea at PCT-level are associated with measures of disadvantage as defined by Indices of Multiple Deprivation and Child Well-being and whether there is variation by length of stay and age.

## Methods

The design incorporated a secondary analysis of Hospital Episode Statistics (HES) and the most contemporaneous Index of Multiple Deprivation (IMD) 2007 and Local Index of Child Well-being (CWI) 2009.

HES is a national data warehouse containing details of admissions to National Health Service (NHS) hospitals in England and NHS commissioned activity in the independent healthcare sector. HES data are recorded as Finished Consultant Episodes (FCEs) which represent a period of admitted patient care under a consultant within an NHS Trust. FCEs are not analogous to a single stay (spell) in hospital because a patient may transfer between two consultants during their stay. FCEs (n = 23,955) were therefore linked to create spells (n = 23,496) prior to analysis. Most spells (n = 23,062, 98.1%) comprised a single FCE and a small number (n = 434, 1.9%) included more than one FCE. There were 72 inter-hospital transfers.

HES were obtained for all children aged 0-14 resident in one of 10 adjacent PCTs serving a metropolitan area in North West England discharged during the 2006/07 financial year following emergency admission to NHS hospitals in England. The upper age limit of 15 years was determined by the age bands in which population data are published by the Office for National Statistics (ONS).

Analysis was conducted as part of a larger research project that assessed the costs and effects of different models of Community Children's Nursing Teams (CCNT) that provide alternative care to hospital admission during acute illness [12]. Emergency admissions for the three commonest medical presentations at paediatric EDs (i.e., breathing difficulty, feverish illness and diarrhoea [5-7,13]) were selected for analysis in consultation with local paediatricians as these conditions may be managed at home by CCNTs. Although breathing difficulty, feverish illness and diarrhoea combined account for around half of medical ED attendances [7] these three categories do not however capture the full range of childhood acute illness. International Classification of Disease (Revision 10) (ICD-10) diagnosis codes used to derive these three categories of admission are shown in (Additional file 1: Table S1).

The three categories of admission are not reported separately because children could be diagnosed with a combination of breathing difficulty, feverish illness, and diarrhoea during a single admission. Most admissions (n = 19,376, 82.5%) were classified into only one of the three condition categories (breathing difficulty: n = 11,780, 50.1%; feverish illness: n = 4,473, 19.0%; diarrhoea: n = 3,123, 13.3%). Around one in every six admissions (n = 4,030, 17.1%) included diagnoses which were classified into two condition categories (breathing difficulty and feverish illness: n = 3,361, 14.3%; breathing difficulty and diarrhoea: n = 341, 1.5%; feverish illness and diarrhoea: n = 328, 1.4%). Ninety (0.4%) admissions included diagnoses classified into all three condition categories.

There is a risk of errors persisting even after processing prior to publication of HES data, and these are known to be more frequent in more specific diagnosis codes and to vary between geographical regions (North West England has one of the lowest error rates) [14]. Clinical coding practice may also differ across hospitals. The potential impact of coding error and disparity was minimised by: (1) selecting conditions predominantly at the 'block' level of the ICD-10 coding structure rather than specific diagnostic codes; (2) identifying emergency admissions in each condition category by examining all fourteen available diagnosis fields in HES rather than reliance on the primary diagnosis because symptoms rather than diagnoses may be recorded in this field following initial assessment; (3) aggregating the three condition categories prior to analysis due to co-morbidity.

EARs were calculated as the number of in-patient spells per 1,000 children aged 0-14 resident in each PCT. As HES do not record admission and discharge time it is possible for an overnight stay (with a recorded length of stay of 1 day) to be shorter than a same day discharge (0 day). EARs were therefore disaggregated by length of stay in three groups: 0 or 1 day; 2 or 3 days; 4 or more days. Age groups were constructed to match those reported by ONS mid-2006 population estimates which were used as the denominator: under 1 year; 1-4 years; 5-9 years; 10-14 years. PCTs were selected as the unit of analysis as the contemporary level at which NHS care was commissioned.

The primary measure of deprivation was the published average IMD 2007 score for each PCT obtained from the Department of Communities and Local Government [15].

Secondary measures of childhood disadvantage were selected domains of the CWI 2009 [16,17]. The CWI 2009 includes seven domains combined with equal weights: material, health, education, crime, housing, environment, and children (at risk of being) in need.

Indicators used to derive the material domain included the percentage of children under 16 years old that live in families reliant on means-tested benefits including: Income Support, Income-Based Job Seekers Allowance, Working Tax or Child Tax Credit. The health domain included rates of emergency admission and outpatient attendance for children aged 0-18 years old and the percentage of children aged 0-16 years old in receipt of Disability Living Allowance. The education domain included indicators of educational attainment at Key Stages 2, 3 and 4, secondary school absence rates, and destinations of children at age 16. The crime domain included rates of burglary, theft, criminal damage and violence. Both the housing and environment domains included two equally weighted sub-domains relating to access and quality. Access to housing was indicated by overcrowding, shared accommodation, and homelessness. Quality of housing was indicated by lack of central heating. The environmental access sub-domain included the proximity of sports and leisure facilities within walking distance, and distance to school. Environmental quality was indicated by air quality, the percentage of green space and woodland, the number of bird species, and road safety measured through road traffic accidents. Due to missing data on the numbers of children served by local authorities at small-area level, the children (at risk of being) in need domain was modelled using the material and education domains of the CWI and the income and employment domains of the IMD 2007 [16,17].

The percentage of Lower Super Output Areas (LSOAs, average populations of 1,500) in each PCT that were in the fifth quintile (lowest well-being) of the 32,482 LSOAs in England was calculated for six of the seven domains of the CWI 2009 (material; education; crime; housing; environment; and children (at risk of being) in need). As the health domain includes an EAR of children aged 0-18 in each LSOA as an indicator there is an in-built association with the EAR for children aged 0-14. This domain and the overall CWI measure incorporating this domain have therefore been excluded from our reporting in order to avoid an obvious bias.

Each PCT's 'local hospital' was defined as the hospital to which the largest percentage of children resident in each PCT was admitted.

Data were analysed descriptively using SPSS Release 15. Associations were measured using Kendall's tau_b _correlation due to expectedly skewed distributions. Calculation of associations using Spearman's Rho correlation confirmed the pattern of these results.

The study was assessed as not requiring ethics approval by a NHS Research Ethics Committee.

## Results

In 2006/07 there were 23,496 emergency admissions of children aged 0-14 resident in the study area to hospitals in England, 98% (n = 23,018) of which were to one of the 12 hospitals in the study area with paediatric facilities (Table 1). Almost four-fifths of admissions were for children under 5 (79%, n = 18,539) and just under a third were under 1 (32%, n = 7,542) (Table 2). Three-quarters of admissions were for a duration of 0 or 1 day (75%, n = 17,670) and most of these were discharged on the same day (i.e., 0 day admissions, n = 10,681, 46% of all admissions) (Table 2).

**Table 1:** Emergency admissions from each Primary Care Trust (PCT) to Hospitals across the study area

	Hospital^†^ n (%)													
**PCT^†^**	A*	**B**	**C**	**D**	**E**	**F**	**G**	H*	**I**	**J**	**K**	**L**	**Out of area**	**Total**

**a**	‡	**1,402**	-	367	-	-	-	103	-	-	-	‡	160	2,042
		**(68.7)**		(18.0)				(5.0)					(7.8)	(100)

**b**	‡	‡	‡	**3,067**	-	-	-	82	-	-	‡	‡	33	3,211
				**(95.5)**				(2.6)					(1.0)	(100)

**c**	365	-	‡	44	‡	‡	‡	100	‡	‡	**905**	-	23	1,462
	(25.0)			(3.0)				(6.8)			**(61.9)**		(1.6)	(100)

**d**	385	‡	-	‡	‡	-	‡	74	40	**1,404**	194	-	25	2,132
	(18.1)							(3.5)	(1.9)	**(65.9)**	(9.1)		(1.2)	(100)

**e**	**1,789**	‡	706	‡	‡	‡	1,260	226	‡	‡	‡	‡	97	4,166
	**(42.9)**		(16.9)				(30.2)	(5.4)					(2.3)	(100)

**f**	205	-	‡	-	27	-	‡	49	**2,164**	‡	‡	-	24	2,513
	(8.2)				(1.1)			(1.9)	**(86.1)**				(1.0)	(100)

**g**	137	‡	-	174	-	-	‡	**1,818**	-	‡	-	‡	22	2,188
	(6.3)			(8.0)				**(83.1)**					(1.0)	(100)

**h**	‡	‡	135	-	‡	**2,065**	24	37	-	-	‡	‡	44	2,323
			(5.8)			**(88.9)**	(1.0)	(1.6)					(1.9)	(100)

**i**	58	-	‡	‡	**2,151**	59	‡	40	‡	‡	-	-	23	2,373
	(2.4)				**(90.6)**	(2.5)		(1.7)					(1.0)	(100)

**j**	‡	‡	430	‡	‡	‡	68	54	-	-	-	**492**	27	1,086
			(39.6)				(6.3)	(5.0)				**(45.3)**	(2.5)	(100)

**Total**	2,974	1,416	1,280	3,662	2,211	2,165	1,402	2,583	2,228	1,442	1,127	528	478	23,496
	(12.7)	(6.0)	(5.4)	(15.6)	(9.4)	(9.2)	(6.0)	(11.0)	(9.5)	(6.1)	(4.8)	(2.2)	(2.0)	(100)

Data Source: Copyright ^© ^2008, Hospital Episode Statistics (HES): Health and Social Care Information Centre. All rights reserved.

^† ^Primary Care Trusts and Hospitals have been anonymised using lower and upper case letters respectively to enable cross-referencing to related publications [18].

^‡ ^Admissions comprising < 1% of the total from each PCT have been suppressed to ensure anonymity and improve clarity.

* Specialist children's hospital.

**Table 2:** Emergency admissions by age and length of stay

	Age Group (years) n(%)									
**Length of Stay (days)**	**Under 1**		**1 to 4**		**5 to 9**		**10 to 14**		**0 to 14**^†^	

0 or 1	5,408	(72)	8,616	(78)	2,354	(76)	1,292	(70)	17,670	(75)
2 or 3	1,358	(18)	1,734	(16)	517	(17)	394	(21)	4,003	(17)
4 or more	776	(10)	647	(6)	215	(7)	171	(9)	1,809	(8)

**All**	7,542	(100)	10,997	(100)	3,086	(100)	1,857	(100)	23,482	(100)

Data Source: Copyright ^© ^2008, Hospital Episode Statistics (HES): Health and Social Care Information Centre. All rights reserved

^† ^Total is not identical to Table 1 as length of stay could not be determined for 14 cases

In eight of the ten PCTs in the study area more than 50% of admissions in 2006/07 were to the 'local hospital' (Table 1). The range across PCTs was 62% (PCT c) to 96% (PCT b) (Table 1). In the remaining two PCTs admissions of less than 50% to a single site can be explained by the existence of two within-area hospitals (in the case of PCT e) or the location of a within-area hospital at the edge of the PCT making a second site in a neighbouring PCT more accessible for some of the resident population (PCT j) (Table 1).

The EAR per 1,000 children under 15 years old was 49.4 (Table 3). Children under 1 year old had the highest rate of admission and the highest EAR was for shorter admissions (Table 3). Substantial differences in EARs were evident across PCTs, ranging between 27.9 and 62.7 (Table 3).

**Table 3:** Emergency admissions rates per 1,000 population by Primary Care Trust (PCT) by age and length of stay

		Emergency Admission Rate per 1,000 population				
		**Age (years)**				

**PCT**	**Length of stay (days)**	**Under 1**	**1 to 4**	**5 to 9**	**10 to 14**	**0 to 14**

a	0 or 1	122.2	50.9	9.9	4.9	26.1
	2 or 3	45.7	13.9	3.5	2.3	8.5
	4 or more	15.1	4.5	0.9	0.4	2.6
	
	All	183.0	69.3	14.3	7.6	**37.2**

b	0 or 1	239.4	93.5	21.0	11.5	52.0
	2 or 3	36.1	11.1	3.2	2.2	7.2
	4 or more	21.1	4.7	1.5	1.0	3.5
	
	All	296.7	109.4	25.7	14.7	**62.7**

c	0 or 1	107.0	52.6	11.8	7.0	26.9
	2 or 3	50.4	21.2	3.4	2.9	10.9
	4 or more	27.8	6.6	0.9	1.6	4.4
	
	All	185.2	80.4	16.1	11.4	**42.2**

d	0 or 1	169.0	73.0	18.9	8.9	39.9
	2 or 3	33.1	13.0	4.1	2.7	8.0
	4 or more	24.5	5.0	1.8	1.7	4.2
	
	All	226.6	91.0	24.8	13.2	**52.1**

e	0 or 1	111.8	64.8	15.6	7.1	35.9
	2 or 3	47.2	18.6	4.0	3.0	11.8
	4 or more	31.0	8.4	2.7	1.4	6.5
	
	All	190.0	91.9	22.4	11.6	**54.2**

f	0 or 1	193.8	83.7	18.7	9.0	45.3
	2 or 3	33.8	9.9	3.0	2.0	6.7
	4 or more	21.6	3.7	0.9	1.1	3.1
	
	All	249.1	97.3	22.6	12.0	**55.1**

g	0 or 1	212.8	81.5	15.6	7.8	45.5
	2 or 3	39.0	13.2	3.0	1.5	7.9
	4 or more	29.7	4.6	1.4	1.1	4.3
	
	All	281.4	99.2	20.0	10.4	**57.7**

h	0 or 1	172.9	72.2	16.1	8.8	37.0
	2 or 3	37.4	11.9	3.3	2.2	7.1
	4 or more	19.4	3.1	1.4	0.8	2.7
	
	All	229.7	87.3	20.8	11.7	**46.8**

i	0 or 1	201.9	76.8	18.8	9.1	42.4
	2 or 3	28.1	11.2	3.6	2.7	6.9
	4 or more	14.2	4.7	0.8	1.3	2.9
	
	All	244.2	92.8	23.3	13.2	**52.1**

j	0 or 1	73.7	35.7	6.6	4.4	18.2
	2 or 3	37.8	11.7	2.5	2.3	7.3
	4 or more	14.4	3.6	1.0	0.2	2.4
	
	All	125.9	51.1	10.2	7.0	**27.9**

All	0 or 1	157.7	68.8	15.5	7.9	37.1
	2 or 3	39.6	13.8	3.4	2.4	8.4
	4 or more	22.6	5.2	1.4	1.0	3.8
	
	All	219.9	87.8	20.3	11.3	**49.4**

Data Source: Copyright ^© ^2008, Hospital Episode Statistics (HES): Health and Social Care Information Centre. All rights reserved

The EAR for children under 15 was associated with the IMD 2007 (Kendall's tau_b _= 0.54, *p *= 0.031) and four domains of the CWI: education (Kendall's tau_b _= 0.58, *p *= 0.020); crime (Kendall's = tau_b _0.54, *p *= 0.031); housing (Kendall's tau_b _= 0.54, *p *= 0.031); children in need (Kendall's tau_b _= 0.49, *p *= 0.048) (Table 4).

**Table 4:** Kendall's tau_b _correlations between emergency admission rate (EAR) for children under 15 and children under 1 and Indices of Multiple Deprivation (IMD) 2007 and Child Well-being (CWI) 2009

		IMD	CWI Quintile 5 (lowest well-being)					
		**Average Score**	**Material**	**Education**	**Crime**	**Housing**	**Environment**	**Children in Need (CiN)**

**EAR (0-14 y) **(days)	0 to 3	0.42	0.29	0.47	0.42	0.42	0.11	0.38
		p = 0.089	p = 0.245	p = 0.060	p = 0.089	p = 0.089	p = 0.655	p = 0.128
	4 or more	**0.64**	**0.51**	**0.51**	**0.56**	0.38	**0.60**	**0.51**
		p = 0.009**	p = 0.040*	p = 0.040*	p = 0.025*	p = 0.128	p = 0.016*	p = 0.040*
	All LOS	**0.54**	0.41	**0.58**	**0.54**	**0.54**	0.23	**0.49**
		p = 0.031*	p = 0.106	p = 0.020*	p = 0.031*	p = 0.031*	p = 0.369	p = 0.048*

**EAR (< 1 y) **(days)	0 to 3	0.29	0.16	0.33	0.20	0.20	0.07	0.24
		p = 0.245	p = 0.531	p = 0.180	p = 0.421	p = 0.421	p = 0.788	p = 0.325
	4 or more	**0.60**	0.47	**0.56**	**0.51**	0.16	**0.56**	0.47
		p = 0.016*	p = 0.060	p = 0.025*	p = 0.040*	p = 0.531	p = 0.025*	p = 0.060
	All LOS	0.33	0.20	0.38	0.33	0.33	0.11	0.29
		p = 0.180	p = 0.421	p = 0.128	p = 0.180	p = 0.180	p = 0.655	p = 0.245

*** *p *< 0.001, ** *p *< 0.01, * *p *< 0.05

Data Source: Copyright ^© ^2008, Hospital Episode Statistics (HES): Health and Social Care Information Centre. All rights reserved

There were no statistically significant relationships between shorter (0 to 3 day) length hospital stays and the IMD or any of the reported CWI domains (Table 4; Additional file 1: Table S2).

There was an association between the EAR for longer (4 or more day) admissions and the IMD (Kendall's tau_b _= 0.64, *p *= 0.009) and five of the six reported domains of the CWI: Environment (Kendall's tau_b _= 0.60, *p *= 0.016); Crime (Kendall's tau_b _= 0.56, *p *= 0.025); Material (Kendall's tau_b _= 0.51, *p *= 0.040); Education (Kendall's tau_b _= 0.51, *p *= 0.040); and Children in Need (Kendall's tau_b _= 0.51, *p *= 0.040) (Table 4). This relationship was strongest with the environment domain (Kendall's tau_b _= 0.60, *p *= 0.016) (Table 4).

There were associations between the EAR of children under 1 and the IMD and three domains of the CWI but only for longer (4 or more day) lengths of stay: IMD (Kendall's tau_b _= 0.60, *p *= 0.016); CWI Environment (Kendall's tau_b _= 0.56, *p *= 0.025), Education (Kendall's tau_b _= 0.56, *p *= 0.025), Crime (Kendall's tau_b _= 0.51, *p *= 0.040) (Table 4; Additional file 1: Table 3).

There were observable differences in the length of stay of children admitted to different hospitals from the same PCT (Table 5). In seven PCTs the highest percentage of short stay admissions (0 to 3 day) discharged on the day of admission was more than two times greater than the lowest and in three of these PCTs this ratio was greater than 3, and in one (PCT c) greater than 4 (Table 5). By contrast, there was little variation in the percentage of same day discharges of children resident in different PCTs attending the same hospital: in only one (tertiary children's) hospital (H) was the ratio between the highest and lowest percentage greater than 2 (Table 5).

**Table 5:** Same day discharges by Hospital for each Primary Care Trust (PCT)

	Hospital^†^ n (% of short stay [0 to 3 day] admissions)												
**PCT^†^**	**A^‡^**	**B**	**C**	**D**	**E**	**F**	**G**	**H^‡^**	**I**	**J**	**K**	**L**	**Ratio (Max/Min)**

**a**	-	333	-	213	-	-	-	42	-	-	-	-	2.4
		(25.5)		(60.0)				(51.2)					

**b**	-	-	-	1,759	-	-	-	31	-	-	-	-	1.1
				(60.2)				(55.4)					

**c**	225	-	-	31	-	-	-	58	-	-	128	-	4.5
	(67.0)			(72.1)				(65.9)			(16.1)		

**d**	222	-	-	-	-	-	-	28	17	717	33	-	3.5
	(66.1)							(50.9)	(44.7)	(54.1)	(19.1)		

**e**	1,086	-	116	-	-	-	229	72	-	-	-	-	3.7
	(67.6)		(18.3)				(21.0)	(45.3)					

**f**	117	-	-	-	12	-	-	9	1,010	-	-	-	2.1
	(64.6)				(57.1)			(31.0)	(48.5)				

**g**	82	-	-	88	-	-	-	1,095	-	-	-	-	1.3
	(67.8)			(52.7)				(64.9)					

**h**	-	-	28	-	-	987	7	18	-	-	-	-	3.3
			(22.6)			(50.2)	(36.8)	(75.0)					

**i**	34	-	-	-	1,241	32	-	14	-	-	-	-	1.3
	(73.9)				(60.5)	(56.1)		(60.9)					

**j**	-	-	76	-	-	-	14	28	-	-	-	100	3.2
			(19.2)				(23.0)	(62.2)				(22.2)	

**Ratio **(Max/Min)	1.1	-	1.2	1.4	1.1	1.1	1.8	2.4	1.1	-	1.2	-	-

Data Source: Copyright ^© ^2008, Hospital Episode Statistics (HES): Health and Social Care Information Centre. All rights reserved.

^† ^Primary Care Trusts and Hospitals have been anonymised using lower and upper case letters respectively to enable cross-referencing to related publications [18]. ^‡ ^Specialist children's hospital.

## Discussion

The rate of emergency admission for breathing difficulty, feverish illness or diarrhoea varied widely across the PCTs in the study from 27.9 to 62.7 per thousand. The overall EARs were associated with deprivation measured by the IMD 2007 and the crime, children in need, and education domains of the CWI 2009 measured at PCT-level. However the relationship between deprivation and EARs was more complicated when children's age and length of stay in hospital were taken into account. The EAR for longer (4 or more day) admissions was associated with the IMD 2007 and the environment, crime, children in need, education and material domains of the CWI 2009 measured at PCT-level. Children living in more deprived areas were at higher risk of a longer hospitalisation which suggests an association between deprivation and illness severity contributing to variations between areas of population. Children in families with lower incomes have poorer health [19] and socio-economic factors are important predictors of hospitalisation although it is not clear how socio-economic disadvantage causes or accentuates childhood ill health [9,20] and the direct effect is reduced when factors that may mediate the impact of income on health are taken into account [21]. The relationship between material deprivation and children's health is complex because financial resources do not straightforwardly buy health, although higher incomes facilitate access to better housing quality and location that may mitigate illness susceptibility and severity. Potential causes of increased illness severity and the risk of prolonged hospitalisation for breathing difficulty include indoor and outdoor environmental exposures such as passive cigarette smoke [22,23] and traffic-related air pollutants [24] which may be higher in deprived areas. Interventions to improve housing can reduce hospitalisation rates for children as well as adults [25]. Environmental conditions including air quality and access to play spaces may be part of the mechanism by which socio-economic disadvantage influences children's respiratory health [26]. Thus there is potential for children's health to be affected by area-level factors such as the accessibility of sports and leisure facilities, open green space including gardens, parks and the natural environment. Such area-level effects are consistent with the finding in this study of strong association between longer lengths of stay and the environment domain of the CWI.

However, admissions of 4 or more days represented only 8% of the dataset, and the EARs for unplanned short stay hospitalisations of less than 4 days (92% of all admissions) were not statistically significantly related to multiple deprivation or any aspect of childhood disadvantage at PCT-level.

For children under 1 year of age only hospitalisations of at least 4 days were associated with the IMD, education, crime and environment domains of the CWI (although children in need and material domains approached statistical significance with weaker correlations) (Table 4). A study of EARs in Greater London found that they were not associated with area-based measures of deprivation in children aged under 1 year [8]. Our findings build on this work by including information on length of stay and demonstrating association with longer but not shorter admissions among infants under 1 year old.

The lack of statistically significant relationships between shorter (0 to 3 day) admissions and measures of deprivation and well-being at PCT-level may be explained by parental help-seeking and professionals' admission decision-making behaviour, particularly for children aged under 1 year. These findings and the high rate of admission of younger children are consistent with parents seeking help due to anxieties about acute illness in young children [27], the threshold for which may be low across all deprivation groups. Non-specific acute illness is common, particularly in infancy, and may lead to caution among healthcare professionals around admission.

Service organisation may also explain variation in EARs at PCT-level of 0 to 3 days not associated with deprivation, especially given the primacy of the 'local hospital' in each PCT. It has been suggested that increases in unplanned short stay admissions may indicate a failure of primary care services which in the past could have managed these children at home [3]. Admissions via EDs increased by 30% between 2002/03 and 2006/07 [1] which could suggest that more children bypassed their General Practitioners (GP) to attend EDs. However, there is also potential for effects to result from changes in hospital care, including the introduction of units for short term observation and assessment of children with medical conditions [28]. There were substantial variations between hospitals in the proportion of children discharged on the day of admission, suggesting differences in their admission and discharge policies. It is known that higher numbers of annual discharges are related to the proportion of same day discharges at local hospitals across the study area [18]. Therefore PCT EARs could be affected by local policies on criteria for admission and management of demand. Because most children attended their 'local hospital' there is limited potential for cross PCT comparison, but where children from the same PCT attended different hospitals there was much greater variation in same day discharge than for children from different PCTs attending the same hospital. There was notable consistency of proportion of same day discharge at Hospitals A (children admitted from 6 PCTs), Hospital C (2 PCTs), and variation in same day discharge rates at different hospitals admitting children from PCTs a, c, e, and f (Table 5). Further research is required to establish whether differences in hospitals' admission and discharge practices can explain the variation in EARs that are not associated with deprivation at PCT-level. It is possible that associations between deprivation and emergency admission exists at a smaller scale and that these 'hospital-level' effects mask relationships with deprivation when assessed at PCT-level.

It is also important to consider the urgent care system as a whole because hospital admissions can be affected by the accessibility and organisation of other services commissioned by PCTs including 'out of hours' GPs [3]. Thus hospitals could have developed observation and assessment services that enable same day discharge in response to demand because children seek help at their local hospital rather than their GPs. It is also possible that parents could choose to attend such services rather than attending a GP who may not have specialist paediatric knowledge, leading to more hospital based care of children with self-limiting illnesses.

### Strengths and limitations

This is the first study to our knowledge to examine associations between children's EARs and measures of childhood disadvantage at PCT-level for different lengths of stay. The main limitation of the study design is the unit of analysis. Although PCT-level analysis is appropriate to ensure findings inform commissioning decisions extrapolation of reported results to individuals risks the ecological fallacy. Both the IMD and CWI are aggregate measures that can hide within-area variation and identify socio-economic and other conditions affecting a group of people by where they live rather than their individual circumstances. Further loss of discrimination is inevitable when these indicators are assessed at PCT-level. Calculation of EARs is also contingent upon accurate HES data and ONS mid-year population estimates which are based on estimation of changes since the 2001 census. In addition, the power of the study was limited because of the number of PCTs included (n = 10). Power could be increased in a future study by examination of EARs at a lower level of geography or in a larger number of PCTs. As EARs are aggregate-level data further research using individual-level analysis would be required to enable adjustment for potential confounding variables.

## Conclusions

Only longer (4 or more day) lengths of stay were associated with population-level measures of multiple deprivation and child well-being, which suggests that deprivation adversely affects illness severity. Material resources may ameliorate illness severity through access to areas with better environmental quality. Although caution must be exercised due to the potential for within-area variation, these findings could be used by commissioners and local authorities (whose boundaries across the study area are largely coterminous) to identify areas where resources could be redirected to develop interventions to improve environmental quality and access to reduce the risk of prolonged hospitalisation, particularly among infants.

The majority of admissions were for shorter hospitalisations which were not associated with deprivation and well-being. It may be that parental anxiety, professionals' admission decisions, local hospital admission and discharge policies, and service organisation masks variation in rates of emergency admission associated with deprivation when analysed at PCT-level. Further research is required using a lower level of geography or at an individual-level to test this hypothesis and determine whether the reported findings remain evident at a smaller scale.

## Competing interests

The authors declare that they have no competing interests.

## Authors' contributions

RGK obtained HES and secondary data, designed and conducted data analysis, conducted interpretation, drafted and revised the manuscript. MC helped shape the analysis and interpretation, commented on revisions to the manuscript. PP aided with interpretation and commented on the manuscript. PC shaped the analysis and interpretation, drafted and revised the manuscript. All authors read and approved the final version.

## Pre-publication history

The pre-publication history for this paper can be accessed here:

http://www.biomedcentral.com/1471-2431/12/22/prepub

## Supplementary Material

Additional file 1 **Tables S1**. ICD codes and descriptors used to identify three commonest reasons for emergency hospital admission. **Table S2**. Kendall's tau_b _correlations between emergency admission rate (EAR) for children under 15 and Indices of Multiple Deprivation (IMD) 2007 and Child Well-being (CWI) 2009. **Table S3**. Kendall's tau_b _correlations between emergency admission rate (EAR) for children under 1 and Indices of Multiple Deprivation (IMD) 2007 and Child Well-being (CWI) 2009. Click here for file
