# Supplementary material for: Relationships between deprivation and duration of children's emergency admissions for breathing difficulty, feverish illness and diarrhoea in North West England: an analysis of hospital episode statistics
Source: BMC Pediatr. 2012 Mar 8;12:22. doi: 10.1186/1471-2431-12-22 (PMC3311147; doi:10.1186/1471-2431-12-22)
Supplement: Additional file 1 — Tables S1. ICD codes and descriptors used to identify three commonest reasons for emergency hospital admission. Table S2. Kendall's taub correlations between emergency admission rate (EAR) for children under 15 and Indices of Multiple Deprivation (IMD) 2007 and Child Well-being (CWI) 2009. Table S3. Kendall's taub correlations between emergency admission rate (EAR) for children under 1 and Indices of Multiple Deprivation (IMD) 2007 and Child Well-being (CWI) 2009. [file 1471-2431-12-22-S1.DOC]

**Additional Material File**

**Table S1**

ICD codes and descriptors used to identify three commonest reasons for emergency hospital admission

| ICD-10 code | | ICD-10 descriptor |
| --- | --- | --- |
| **Breathing Difficulties** | | |
| A37 | Whooping cough | |
| J00-J06 | Acute upper respiratory infections | |
| J09-J16 | Influenza and pneumonia | |
| J18 | Pneumonia, organism unspecified | |
| J20-J22 | Other acute lower respiratory infections | |
| J30-J39 | Other diseases of upper respiratory tract | |
| J40-J46 | Chronic lower respiratory diseases | |
| J68-J69 | Lung diseases due to external agents | |
| J96 | Other respiratory disorders | |
| J98 | Respiratory failure, not elsewhere classified | |
| R05 to R06 | Cough; Abnormalities of breathing | |
|  |  | |
| **Diarrhoea** | | |
| A00 | Intestinal infectious diseases | |
| A02 to A09 | Intestinal infectious diseases | |
| A32 | Listeriosis | |
| K52.9 | Noninfective gastroenteritis and colitis, unspecified | |
|  |  | |
| **Feverish Illnesses** | | |
| A01 | Typhoid and paratyphoid fevers | |
| A20 to A28 | Certain zoonotic bacterial diseases | |
| A38 | Scarlet fever | |
| A48 | Other bacterial diseases, not elsewhere classified | |
| A49 | Bacterial infection of unspecified site | |
| B01.2 | Varicella and pneumonia | |
| B01.8 | Varicella with other complications | |
| B01.9 | Varicella without complication Varicella NOS | |
| B05.8 | Measles with other complications; Measles keratitis and keratoconjunctivitis | |
| B05.9 | Measles without complication; Measles NOS | |
| B06.8 | Rubella with other complications; arthritis; pneumonia | |
| B06.9 | Rubella without complication; Rubella NOS | |
| B26.8 | Mumps with other complications; arthritis; myocarditis; nephritis; polyneuropathy | |
| B26.9 | Mumps without complication Mumps: NOS; parotitis NOS | |
| B27 | Infectious mononucleosis | |
| B34 | Viral infection of unspecified site | |
| B34.8 | Other viral infections of unspecified site | |
| B34.9 | Viral infection, unspecified | |
| B99 | Other infectious diseases | |
| H65.0 | Acute serous otitis media | |
| H66.0 | Acute suppurative otitis media | |
| H66.9 | Otitis media, unspecified | |
| R50 | Fever of other and unknown origin | |
| N39.0 | Urinary tract infection, site not specified | |

**Table S2**

Kendall’s tau_b_ correlations between emergency admission rate (EAR) for children under 15 and Indices of Multiple Deprivation (IMD) 2007 and Child Well-being (CWI) 2009

|  |  | **EAR 2006/07**  (days) | | | | | **IMD** |  | **CWI Quintile 5 (lowest well-being)** | | | | |
| --- | --- | --- | --- | --- | --- | --- | --- | --- | --- | --- | --- | --- | --- |
|  |  | 0 or 1 | 2 or 3 | *0 to 3* | 4 or more | All LOS | Average Score | Material | Education | Crime | Housing | Environ-ment | Children in Need (CiN) |
| **EAR** (days) | 0 or 1 | – | -0.33  p=0.180 | *0.96*  *p<0.001**** | 0.29  p=0.245 | 0.85  P=0.001** | 0.38  p=0.128 | 0.24  p=0.325 | 0.42  p=0.089 | 0.38  p=0.128 | 0.38  p=0.128 | 0.07  p=0.788 | 0.33  p=0.180 |
|  | 2 or 3 |  | – | *-0.29*  *p=0.245* | 0.38  p=0.128 | -0.18  p=0.472 | 0.11  p=0.655 | 0.07  p=0.788 | 0.07  p=0.788 | 0.02  p=0.929 | -0.16  p=0.531 | 0.24  p=0.325 | -0.02  p=0.929 |
|  | *0 to 3* |  |  | *–* | *0.33*  *p=0.180* | *0.90*  *p<0.001**** | *0.42*  *p=0.089* | *0.29*  *p=0.245* | *0.47*  *p=0.060* | *0.42*  *p=0.089* | *0.42*  *p=0.089* | *0.11*  *p=0.655* | *0.38*  *p=0.128* |
|  | 4 or more |  |  |  | – | 0.45  p=0.072 | 0.64  p=0.009** | 0.51  p=0.040* | 0.51  p=0.040* | 0.56  p=0.025* | 0.38  p=0.128 | 0.60  p=0.016* | 0.51  p=0.040* |
|  | All LOS |  |  |  |  | – | 0.54  p=0.031* | 0.41  p=0.106 | 0.58  p=0.020* | 0.54  p=0.031* | 0.54  p=0.031* | 0.23  p=0.369 | 0.49  p=0.048* |
| **IMD** | Average Score |  |  |  |  |  | _ | 0.87  P<0.001*** | 0.87  P<0.001*** | 0.73  p=0.003** | 0.38  p=0.128 | 0.33  p=0.180 | 0.87  P<0.001*** |
| **CWI** | Material |  |  |  |  |  |  | – | 0.73  p=0.003** | 0.69  p=0.006** | 0.33  p=0.180 | 0.29  p=0.245 | 0.82  p=0.001** |
|  | Education |  |  |  |  |  |  |  | – | 0.69  p=0.006** | 0.33  p=0.180 | 0.29  p=0.245 | 0.82  p=0.001** |
|  | Crime |  |  |  |  |  |  |  |  | – | 0.47  p=0.060 | 0.51  p=0.040* | 0.78  p=0.002** |
|  | Housing |  |  |  |  |  |  |  |  |  | – | 0.07  p=0.788 | 0.24  p=0.325 |
|  | Environment |  |  |  |  |  |  |  |  |  |  | – | 0.38  p=0.128 |
|  | CiN |  |  |  |  |  |  |  |  |  |  |  | – |

*** p<0.001, ** p<0.01, * p<0.05

Data Source: Copyright © 2008, Hospital Episode Statistics (HES): Health and Social Care Information Centre. All rights reserved.

**Table S3**

Kendall’s tau_b_ correlations between emergency admission rate (EAR) for children under 1 and Indices of Multiple Deprivation (IMD) 2007 and Child Well-being (CWI) 2009

|  |  | **EAR 2006/07**  (days) | | | | | **IMD** |  | **CWI Quintile 5 (lowest well-being)** | | | | |
| --- | --- | --- | --- | --- | --- | --- | --- | --- | --- | --- | --- | --- | --- |
|  |  | 0 or 1 | 2 or 3 | *0 to 3* | 4 or more | All LOS | Average Score | Material | Education | Crime | Housing | Environ-ment | Children in Need (CiN) |
| **EAR** (days) | 0 or 1 | – | -0.42  p=0.089 | *1.00*  *p<0.001**** | -0.22  p=0.929 | 0.87  P<0.001*** | 0.29  p=0.245 | 0.16  p=0.531 | 0.33  p=0.180 | 0.20  p=0.421 | 0.20  p=0.421 | 0.07  p=0.788 | 0.24  p=0.325 |
|  | 2 or 3 |  | – | *-0.42*  *p=0.089* | 0.24  p=0.325 | -0.29  p=0.245 | -0.07  p=0.788 | -0.11  p=0.655 | -0.22  p=0.929 | -0.24  p=0.325 | -0.24  p=0.325 | 0.16  p=0.531 | -0.20  p=0.421 |
|  | *0 to 3* |  |  | *–* | *-0.02*  *p=0.929* | *0.87*  *p<0.001**** | *0.29*  *p=0.245* | *0.16*  *p=0.531* | *0.33*  *p=0.180* | *0.20*  *p=0.421* | *0.20*  *p=0.421* | *0.07*  *p=0.788* | *0.24*  *p=0.325* |
|  | 4 or more |  |  |  | – | 0.11  p=0.655 | 0.60  p=0.016* | 0.47  p=0.060 | 0.56  p=0.025* | 0.51  p=0.040* | 0.16  p=0.531 | 0.56  p=0.025* | 0.47  p=0.060 |
|  | All LOS |  |  |  |  | – | 0.33  p=0.180 | 0.20  p=0.421 | 0.38  p=0.128 | 0.33  p=0.180 | 0.33  p=0.180 | 0.11  p=0.655 | 0.29  p=0.245 |
| **IMD** | Average Score |  |  |  |  |  | _ | 0.87  P<0.001*** | 0.87  P<0.001*** | 0.73  p=0.003** | 0.38  p=0.128 | 0.33  p=0.180 | 0.87  P<0.001*** |
| **CWI** | Material |  |  |  |  |  |  | – | 0.73  p=0.003** | 0.69  p=0.006** | 0.33  p=0.180 | 0.29  p=0.245 | 0.82  p=0.001** |
|  | Education |  |  |  |  |  |  |  | – | 0.69  p=0.006** | 0.33  p=0.180 | 0.29  p=0.245 | 0.82  p=0.001** |
|  | Crime |  |  |  |  |  |  |  |  | – | 0.47  p=0.060 | 0.51  p=0.040* | 0.78  p=0.002** |
|  | Housing |  |  |  |  |  |  |  |  |  | – | 0.07  p=0.788 | 0.24  p=0.325 |
|  | Environment |  |  |  |  |  |  |  |  |  |  | – | 0.38  p=0.128 |
|  | CiN |  |  |  |  |  |  |  |  |  |  |  | – |

*** p<0.001, ** p<0.01, * p<0.05

Data Source: Copyright © 2008, Hospital Episode Statistics (HES): Health and Social Care Information Centre. All rights reserved.
